# Supported Biofilms on Carbon–Oxide Composites for Nitrate Reduction in Agricultural Waste Water

**DOI:** 10.3390/molecules26102987

**Published:** 2021-05-18

**Authors:** M. Isidora Bautista-Toledo, Francisco J. Maldonado-Hódar, Sergio Morales-Torres, Luisa M. Pastrana-Martínez

**Affiliations:** Department of Inorganic Chemistry, Faculty of Sciences, University of Granada, Avda, Fuente Nueva s/n, 18071 Granada, Spain; bautista@ugr.es (M.I.B.-T.); semoto@ugr.es (S.M.-T.); lpastrana@ugr.es (L.M.P.-M.)

**Keywords:** biofilms, carbon–oxide composites, *Escherichia coli*, *Vibrio fischeri*, denitrification, water treatment

## Abstract

*Escherichia coli* colonies were grown on different supports for the removal of nitrates from water. A carbon material and different commercial metal oxides, such as SiO_2_, TiO_2_ and Al_2_O_3_, and their corresponding carbon–metal oxide composites were studied. The physicochemical properties were analyzed by different techniques and the results were correlated with their performance in the denitrification process. Developed biofilms effectively adhere to the supports and always reach the complete reduction of nitrates to gaseous products. Nevertheless, faster processes occur when the biofilm is supported on mesoporous and non-acid materials (carbon and silica).

## 1. Introduction

Water pollution, involving both organic and inorganic pollutants, is a major concern in different areas worldwide. The concentration and nature of pollutants are essentially related to human activities such as residence, industry, agriculture, etc. The concentrations of many different organic products, including emerging and recalcitrant drugs, personal care products (PCPs) and dyes from textile industries, are progressively increasing in water resources. A large variety of experimental procedures based on advanced oxidation processes (AOPs), biological treatments, membrane separation or adsorption procedures, or even, their combinations, have been developed to try to remove or mineralize these organic pollutants [1,2,3,4]. Mineralization is always desirable, avoiding the formation of residues, sludge and intermediate organic compounds, which can be even more dangerous than the original pollutants. Similarly, the pollution of water by inorganic compounds, such as heavy metals from mining or nitrates and/or phosphates mainly from agricultural activities, causes not only different alterations on human health, but also additional problems such as the eutrophication of lakes and water reservoirs [5,6].

Among others, nitrates in groundwater are typically associated with intensive agricultural environments using large amounts of fertilizers and can produce many diseases, including gastric cancer [7]. In addition, nitrates are very soluble and stable, and easily drain through the soil, flowing with the groundwater and accumulating in water reservoirs. On such a basis, the concentration of nitrates and derivative nitrites was limited to 50 mg L**^−^**^1^ and 10 mg L**^−^**^1^, respectively [8]. The reduction of nitrates is today an interesting research field that uses catalytic processes, adsorption, membrane separation or ion exchange [9]. In the case of membrane-driven separation processes, the main drawback is associated with the expensive and concentrated brines generated that should be treated separately [10]. The biodegradation of nitrates by anaerobic bacteria is an interesting alternative [11]; this technology can be combined for the production of electric energy in microbial fuel cell [12].

Independent of the decontamination approach, it is clear that the characteristics of materials used as catalysts, adsorbents, membranes or bacteria supports should be progressively improved in order to increase their performance in the corresponding process. Researchers have focused on the optimization of different materials to be used as bacteria supports for nitrate reduction. Thus, the nature and morphology of these supports largely influenced the bioreactors or constructed wetlands for water treatments [13,14]. Materials with very different properties and compositions were studied as bacteria supports, in particular, zeolites [15,16], fly ash [17], metal oxides [18,19], wood [20], microalgae [21] or activated carbons (ACs) [22]. ACs are typically used for water treatment due to their non-toxic character, low price and mainly, the easy fitting of their porous and chemical properties [23].

However, a large number of novel carbon materials (i.e., graphene, carbon nanotubes, fullerenes, etc.) have emerged with better performance in water treatment processes [24,25]. Among them, carbon gels offer the same facilities of large porosity and controllable surface chemistry, but also additional advantages such as a total purity, homogeneity or the possibility to be prepared with optimized composition, nanostructure, shape or dimension [26,27]. Our research group previously reported the performance of inorganic metal oxides, zeolites and carbon coatings [22,28] as *Escherichia coli* (*E. coli*) bacteria supports for water denitrification. In the case of inorganic metal oxides and zeolitic materials, it was detected that the bacterial activity of denitrification decreased with the increasing acidity of the zeolitic support, leading to a marked delay in intermediate nitrite degradation. However, bacteria growth was favored by enhancing the acid character of the films, when using pure carbon xerogels obtained from a low carbonization degree [29]. In previous works, it was also previously detected that the performance of ACs as bacteria supports depended on the presence of inorganic impurities and large macropores [22].

Therefore, the nature and physicochemical properties of the supports have a significant effect on the development of bacteria colonies and finally, on their activity for the removal of inorganic pollutants. The aim of this study was to establish and elucidate the relationships between the properties and activity of the respective materials. Thus, a series of carbon–metal oxide (C/SiO_2_, C/Al_2_O_3_ or C/TiO_2_) composites were prepared and compared with pure carbon gels or commercial metal oxides, in order to establish the influence of the nature of inorganic phases. The physicochemical properties of these supports were analyzed and correlated with their ability to develop *E. coli* biofilms on their surface. The performance of these bacteria colonies was assessed in the reduction of both nitrates and nitrites in simulated agricultural wastewater; the synergistic effect between carbon and inorganic phases was also analyzed.

## 2. Results and Discussion

Preliminary experiments carried out in the absence of bacteria pointed out that nitrate adsorption or degradation by the supports do not occur, regardless of the material used, with the nitrate concentration remaining stable and the formation of nitrites or other degradation by-products not being detected. Therefore, the decay in nitrate concentration observed after immobilizing the biofilms on the supports is only due to the bacterial activity during the reduction process.

**Figure 1:**
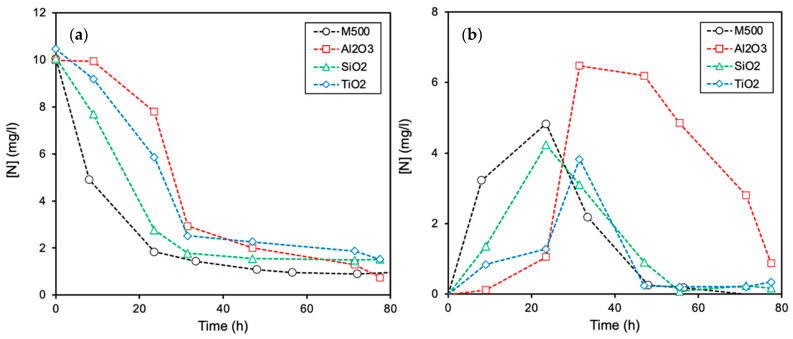
Denitrification activity obtained by *E. coli* colonies deposited on a carbon material (M500) and commercial metal oxides: (**a**) removal of nitrates and (**b**) formation and reduction of nitrites. Data are given as total nitrogen concentration.

Figure 1a shows the biofilm’s performance in the removal of nitrates when supported on pure carbon (i.e., M500) and metal oxide phases (i.e., Al_2_O_3_, SiO_2_ and TiO_2_). Under these experimental conditions, bacteria breathe using nitrates or nitrites as terminal electron acceptors, with denitrification occurring through the transformation of nitrates from contaminated waters to gaseous products. The reduction of nitrates by bacteria occurs following a four-step process [28,30]: microorganisms reduce nitrates (NO_3_^−^) progressively to nitrites (NO_2_^−^), nitric oxide (NO), nitrous oxide (N_2_O), and finally, to nitrogen gas (N_2_). In our case, analysis of the gases was not performed, following only the evolution of the main species in the solution, i.e., NO_3_^−^ and NO_2_^−^. Thus, the formation and degradation of the intermediate nitrite on the different biofilms were also analyzed and compared in Figure 1b. In general, *E. coli* colonies are able to totally mineralize the initial NO_3_^−^ concentration (Figure 1a). The intermediate NO_2_^−^ progressively appears in the solution, as the NO_3_^−^ molecules are reduced, and finally, they are also completely removed (Figure 1b).

At a glance, it is pointed out that the biofilms formed on a carbon phase (M500) are more active than those obtained on inorganic oxides. The reduction of both NO_3_^−^ and NO_2_^−^ concentrations is faster in the trend: Al_2_O_3_ < TiO_2_ < SiO_2_ < M500. This is an important finding, taking into account that ACs are typically used in wastewater treatment plants (WWTPs), produced from different origins/treatments and, therefore, contain different ash (inorganic) content and nature, which can influence their performance. On such a basis, a series of pure carbon–metal oxide composites were prepared in order to elucidate the role of the typical inorganic components on the denitrification process.

**Figure 2:**
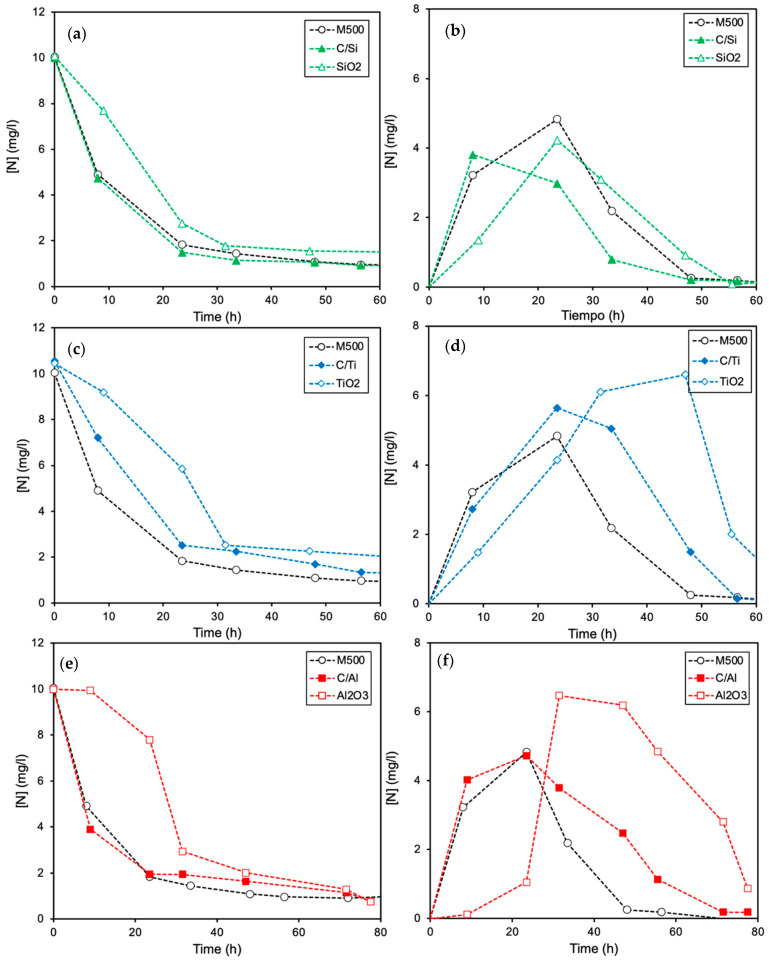
Comparison of the denitrification activity obtained by *E. coli* colonies deposited on carbon–metal oxide composites and their corresponding pure phases: (**a**,**c**,**e**) removal of nitrates and (**b**,**d**,**f**) formation and reduction of nitrites. Data are given as total nitrogen concentration.

The performance of the biofilms supported on the composites regarding their pure phases is compared in Figure 2. As with the case of pure phases, both nitrates and intermediate nitrites are completely removed regardless the material tested, but bacteria supported on the carbon–metal composites present an intermediate performance between the pure carbon and the corresponding metal oxide phase. The reduction processes are progressively slower for composites containing C/SiO_2_, followed by C/TiO_2_ and finally, C/Al_2_O_3_. Thus, the bacterial activity follows the same tendency previously observed by using pure metal oxides (Figure 1). The concentration of intermediate NO_2_^−^ typically decreases after total NO_3_^−^ reduction is achieved (Figure 2b), indicating a preferential reduction for the NO_3_^−^ species.

**Figure 3:**
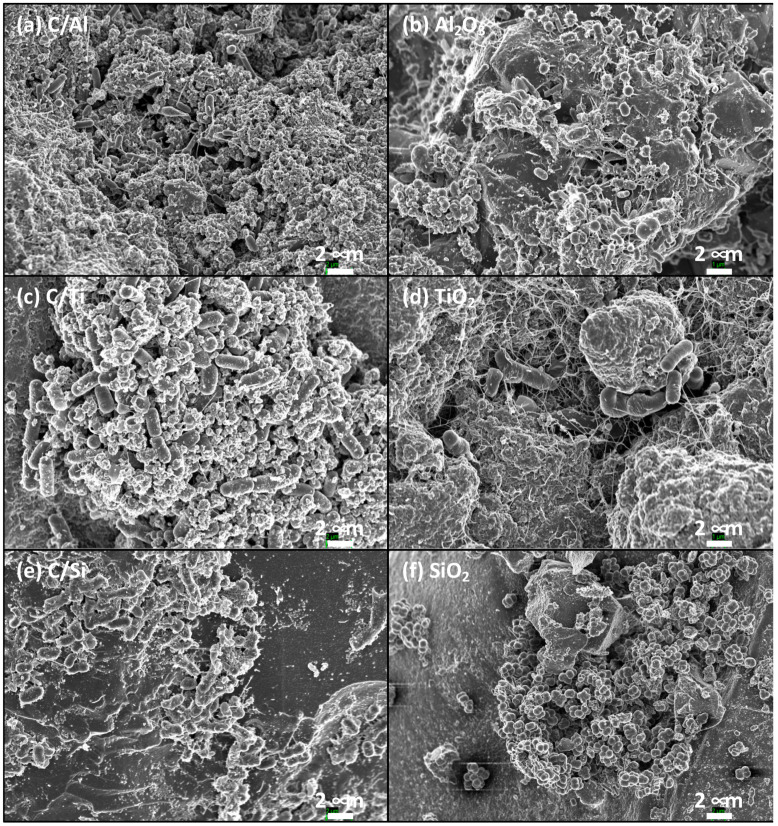
SEM analysis of the biofilms supported on carbon–metal oxide composites (**a**,**c**,**e**), regarding their corresponding pure oxides (**b**,**d**,**f**): (**a**) C/Al_2_O_3_ vs. (**b**) Al_2_O_3_; (**c**) C/TiO_2_ vs. (**d**) TiO_2_; and (**e**) C/SiO_2_ vs. (**f**) SiO_2_.

The morphology of the supported biofilms was analyzed by SEM (Figure 3). In general, the formation of well-adhered *E. coli* colonies was achieved on all the support surfaces. An important factor that could provide additional information about the environment of the biofilm is the secretion of extracellular polymeric substances (EPS). The formation of EPS involves different mechanisms and is influenced by different factors, such as the type of substrate, the nutrient content or the external conditions [31]. In fact, it was suggested that bacteria would excrete more EPS under unfavorable conditions.

The formation of EPS strongly influences the performance of the biofilm in wastewater treatment, in particular the mass transfer and fouling of the membranes used for water treatments [31]. In addition, the EPS structure contains many different functional groups (e.g., carboxylic acid, phenol, hydroxyl, etc.), which induce changes in the interactions with the substances (pollutants) present in water. For instance, EPS determine the nature of the aggregates during the flocculation process when used multivalent metals, as Al^3+^ [32], and enhance the adsorption capacity of species in solution as heavy metals [33] or organic compounds [34,35].

In our case, the formation of EPS seems to be favored when used pure inorganic supports (Figure 3b,d,f), in particular for pure Al_2_O_3_ and TiO_2_ as bacteria supports, regarding their corresponding composites (Figure 3a,c,e). Since the denitrification experiments were carried out under the same experimental conditions, only by changing the bacteria support, it seems that the presence of carbon in the composites clearly reduces the EPS formation, which could suggest a more “friendly” interaction between the bacteria and support. Nevertheless, thermophilic bacteria simultaneously accumulate EPS during the nitrate reduction [36], with a positive correlation between the EPS production and the nitrate removal efficiency.

This different interaction between supports and bacteria can be correlated with the toxicity obtained for the materials. Thus, the toxicity of the supports was measured based on the inhibition degree achieved using the photoluminescent *Vibrio fischeri* bacteria; the results are summarized in Table 1. The toxicity of the pure supports increases in the sense M500 ≈ SiO_2_ < TiO_2_ < Al_2_O_3_, and the same trend is also observed for the corresponding composites, the pure carbon M500 being the least toxic material. The presence of carbon in the composites allows the toxicity of supports to decrease regarding their pure oxides, with the exception of C/SiO_2_, whose toxicity (I_15_ = 8.4%) presents the same order as carbon and silica phases (I_15_ = 7.1%, Table 1). It is noteworthy that the toxicity trend determined for the materials follows the same order in denitrification activity, previously pointed out in Figure 1 and Figure 2.

Taking into account the previous results, an exhaustive characterization of the samples was carried out in order to identify the physicochemical properties of the supports influencing the activity of the biofilms in the anaerobic denitrification process. The textural and acidic–basic characteristics of the pure supports are summarized in Table 2. Textural characteristics are obtained from analysis of the N_2_-adsorption isotherms. The total pore volume (V_T_) was considered as the volume of N_2_ adsorbed at P/P_0_ = 0.95, and the mesopore volume (V_meso_) from the difference between V_T_ and the volume of N_2_ adsorbed at P/P_0_ = 0.40 (V_micro_), following the Gurvich rule. The pure carbon material presents a more developed porosity than the metal oxide supports, including larger volumes of micro- (V_micro_) and mesopores (V_meso_), and consequently, a higher apparent surface area (S_BET_). The micro-mesoporosity of the pure phases used as bacteria supports decreased in the sense: M500 > SiO_2_ > TiO_2_ > Al_2_O_3_. However, the surface acidity of the supports presents the contrary tendency, thus alumina was the most acidic and least porous sample used, while both carbon and silica phases present high mesopore volumes and practically, a neutral character. Therefore, the performance of *E. coli* biofilms on pure materials (Figure 1) could be favored by increasing the porosity and/or decreasing the acidity of the supports.

The composition, acidity (pHpzc) and textural characteristics of the carbon–metal oxide composites are summarized in Table 3. All the composites present a similar metal oxide content (around 45 ± 3%) determined by weighing the ash content obtained after burning a sample fraction. A more exhaustive textural characterization by combining CO_2_ adsorption and mercury porosimetry experiments was performed for composites due to the complexity of the samples and looking for additional relationships. The determination of macroporosity and large mesopores, poorly analyzed by N_2_ adsorption, becomes important, since the size of bacteria is at the micrometer scale (Figure 3), while the adsorption capacity of the pollutants is related with the narrowest microporosity (micropore diameter < 0.7 nm, not accessible to N_2_ at −196 °C and determined with CO_2_ at 0 °C). This microporosity range (V_CO2_) is negligible in pure inorganic oxides, but is favored with the incorporation of the carbon phase in the corresponding composites. Thus, the micropore volume and the corresponding micropore surface area (S_CO2_) decrease regarding the pure carbon phase, but show similar values between the composites, taking into account that the metal oxide content was comparable.

**Figure 4:**
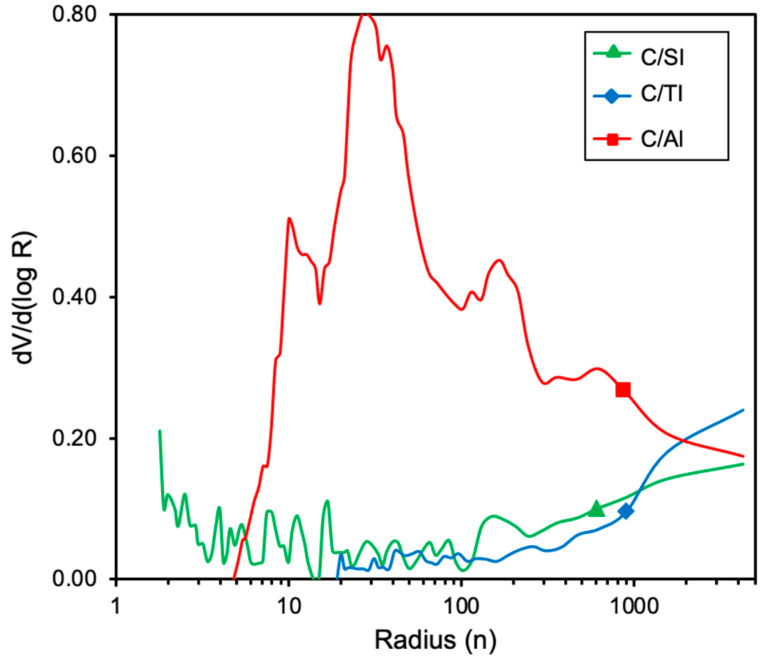
Meso- and macropore size distribution of the carbon–metal oxide composites determined by mercury porosimetry.

The pore size distribution (PSD) determined by mercury porosimetry is plotted in Figure 4, clearly showing a developed macroporosity for C/Al_2_O_3_ regarding the other composites, which also corroborates its significant reduction in density (*ρ*_p,_ Table 3). The volumes of large mesopores (V_meso_) and macropores (V_macro_) for C/Al_2_O_3_ were higher than those for the other composites (Table 3). These results clearly pointed out that the external porosity of the samples is not the limiting factor in the activity of supported biofilms, since bacteria supported on C/Al_2_O_3_ presented the worst denitrification activity (Figure 2), while well-adhered colonies were observed in all cases (Figure 3).

**Figure 5:**
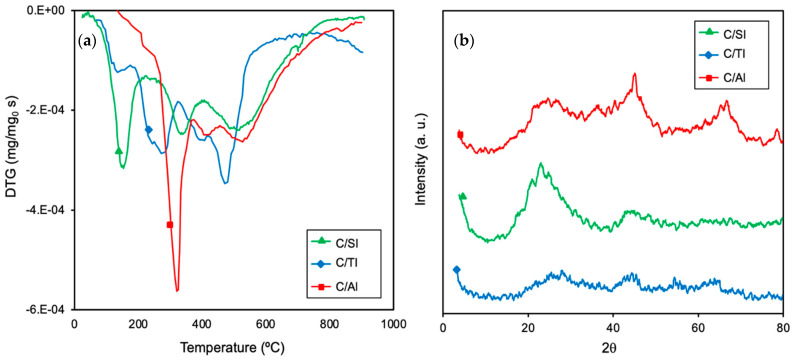
(**a**) Differential thermogravimetric (DTG) profiles obtained for the composites during the carbonization in N_2_ flow at 10 °C min^−1^. (**b**) X-ray diffraction (XRD) patterns obtained for the composites.

Concerning the acidity of the supports, it is noteworthy that the pH_pzc_ of the composites increased regarding the corresponding pure oxides (Table 3 vs. Table 2). This acidity decrease is due to the chemical transformations underwent by both organic and inorganic phases of the samples during the carbonization. The acidic chemical groups are evolved as gases (i.e., CO_x_, H_2_O) according to their thermal stability and the interactions between the metallic phase and the organic matrix. The carbonization process was simulated by thermogravimetric analysis (TG/DTG) and the results are shown in Figure 5a. The DTG profile obtained for the carbonization of pure organic polymers (result not shown for clarity) presents three main peaks centered at around 200 °C, 350 °C and 550 °C, associated with the loss of unreacted products, the breakage of C−O and finally, the reorganization of C−C and C−H bonds [37,38]. The temperature of these DTG peaks in the composites significantly changes between them, indicating different organic polymer–oxide precursor linkages. The marked DTG peaks located at around 320 °C for C/Al_2_O_3_ are worth noting, which could suggest a larger loss of oxygenated surface groups. All composites do not present any significant weight loss from 800 °C, indicating that the main transformations occur at lower temperatures. The intimal interaction between carbon and oxide fractions was also pointed out by X-ray diffraction (XRD) (Figure 5b), with no diffraction peaks being observed that denote the presence of crystalline phases corresponding to the metallic phases, except a small peak at 44.5° in sample C/Al_2_O_3_ that indicates the incipient crystallization of Al_2_O_3_. In other words, the organic phase prevents the crystalline growth of the inorganic phases, even after heat treatment.

Thus, porosity is always large enough, and in spite of the different PSD of the supports, well-adhered colonies of bacteria were observed by SEM. Nevertheless, the performance of these colonies seems to be related with the acidity of the samples. With increasing acidity, specifically the Brönsted acidity predominant on the alumina, the support becomes negatively charged in the buffered (pH = 7) solutions, modifying the interactions with the Gram-negative *E. coli* bacteria, increasing the EPS segregation and decreasing the NO_3_^−^ and more specifically, the NO_2_^−^ reduction.

The development of *E. coli* biofilms on the surface of ACs was studied in previous works [39]. The adsorption of bacteria on ACs modified their surface characteristics, reducing the volume of pores and the pH of the point of zero charge. It was also showed [22] that the macropore volume of granular ACs (GACs) allows bacteria growth during the denitrification process, while the presence of Cd(II) and Cr(VI) in the solution negatively affected the denitrification process kinetics. Nevertheless, electron donor chemicals can also be used to favor nitrate reduction [40]. More recently, it was also demonstrated that *E. coli* biofilms depend on the nature and morphology of the carbon used as the support; activated carbon cloths were a superior adsorbent of *E. coli* compared to GACs, and the presence of bacteria on the carbon materials increased the adsorption of pollutants (bisphenol) between 24% and 33% [41]. Using carbon xerogel films cured by microwave treatments as *E. coli* supports [29], it was observed that low carbonization degree and/or more acidic pH of point of zero charge favor bacteria growth. Nevertheless, when *E. coli* biofilms are supported on alkali or alkali earth exchanged zeolites [28], the acidity of the support clearly retards the reduction of nitrates and, more specifically, nitrite reduction. Others carbon forms also favor the performance of denitrification. The use of micrographite particles demonstrated an increase in abundance of the denitrifying bacteria *Paracoccus* in sludge (not supported), increasing its denitrifying capacity [42]. These results pointed out the importance of the environment on the biofilm’s performance, determined either by the characteristics of the supports or by the presence of other substances in the solution. The different carbon forms available and the versatility of these materials to fit their physicochemical properties, probably also combined with the use of electron donor substances, are interesting routes for the reduction of nitrates in wastewaters.

## 3. Materials and Methods

The performance of different materials as bacteria supports was examined. In particular, a pure carbon gel (sample M500) and carbon–metal oxide composites (C/Si, C/Al and C/Ti) were synthesized by the method developed by Pekala and later, adapted in our laboratories [37,43,44]. A starting solution was prepared by dissolving appropriate amounts of resorcinol (R) and formaldehyde (F) (carbon precursors) in water in a molar ratio of R/F = 1/2 (Sample M). This solution was heated up to 50 °C under vigorous stirring and then, in the case of composites, the corresponding metal alkoxide was dropped at this temperature into the reactor in a resorcinol/alkoxide molar ratio equal to unity. The metal alkoxides used during the synthesis were tetraethyl orthosilicate (TEOS), aluminum isopropoxide (ALPR) and tetrabutyl orthotitanate (TBTi) for the preparation of C/Si, C/Al and C/Ti composites, respectively. The gel formed was redispersed by stirring, aged for 4 h, recovered by filtration, exchanged with acetone and finally, dried for 1 day at 80 °C and 3 days at 110 °C. The pyrolysis of the organic gels was carried out in a tubular furnace using a N_2_ flow of 100 cm^3^ min^−1^, by heating up to 500 °C (sample M500) or 900 °C (composites) with a heating rate of 5 °C min^−1^, and a soaking time of 1.5 h. Commercial metal oxides, i.e., SiO_2_, Al_2_O_3_ and TiO_2_ samples, were used as a reference and without any pretreatment. The oxide metal content of the synthetized samples was determined by burning a fraction of it at 900 °C in air.

Textural characterization of materials was performed using mercury porosimetry (Quantachrome) to determine the macro-mesoporosity (pores greater than 3.6 nm) and physical adsorption of CO_2_ and N_2_ at 0 °C and −196 °C, respectively, to analyze the microporosity. The apparent surface area (S_BET_) was calculated applying the BET equation [45] to N_2_ adsorption isotherms, while Dubinin–Radushkevich and Stoeckli equations [46,47] were used to determine the micropore volume (V_micro_), and the mean micropore width (L_0_). The micropore volume (V_CO2_) and the micropore surface area (S_CO2_) were also estimated from CO_2_ adsorption data. Mesopore volume (V_meso_), macropore volume (V_macro_), bulk density (*ρ*_p_) and external surface area (S_ext_) were calculated by mercury porosimetry. XRD patterns were determined with a BRUKER D8 ADVANCE diffractometer (40 kV and 40 mA) using Cu K_α_ radiation. The point of zero charge (pH_PZC_) of the supports, which is an indication of their surface acidity, was determined by a methodology previously described elsewhere [48,49]. The morphology of supports was characterized by scanning electron microscopy (SEM) using a LEO Carl Zeiss GEMINI-1530. This technique was also used to study the colonization of supports by bacteria after the corresponding denitrification experiments.

Bacteria used in the experiments were *Escherichia coli*, ATCC^®^ 25922^TM^ strain, which was first incubated at 37 °C using a buffered media at pH 7 with tryptic soy broth (TSB), before being immobilized on the different supports. Bacteria were supported on the different solids, adding 1 mL of this suspension to 0.4 g of support suspended in 20 mL of TSB and the mixtures were shaken at 37 °C for 3 days. Afterward, the colonized supports were filtered and washed repeatedly with sterilized distilled water.

The denitrification process of water was studied by bacteria immobilized on the different supports (0.4 g), which were added to 100 mL of a solution containing 50 mg L^−1^ of nitrate from NaNO_3_ and 1.3 mL of ethanol. The suspension was buffered at pH 7 with an appropriate phosphate solution. The flasks used as batch bioreactors were flushed with argon atmosphere to obtain anaerobic conditions and then, placed in a thermostatic rotary shaker at 25 °C. Periodically, the concentration of nitrate was measured directly in the bioreactors with a selective electrode supplied by Mettler, and simultaneously, a small volume of solution (1 mL) was withdrawn for the determination of the nitrite concentration. This parameter was determined using a Hitachi model U2000 spectrophotometer at 543 nm after coupling diazotized sulphanilamide with N-1-naphthyl-ethylendiamine [50].

The toxicity of the inorganic solid suspensions was measured based on the inhibition of the luminescence of *Vibrio fischeri* marine bacteria, NRRL-B-11177. For this, a suspension of bacteria was prepared according to the European guideline ISO 11348-2:2007 and the initial luminescence was determined. The solid supports were removed from the corresponding suspensions by centrifugation to avoid interferences during the determination of the light intensity, and the water recovered was used to dilute the bacterial suspension mixed in a 1:1 ratio. Bioluminescence was measured using a LUMISTOX system after a 15-min exposure. In all measurements, the percentage inhibition (% I) was obtained by comparing the response of a control saline solution with that obtained for the sample. Toxicity was expressed as the percentage inhibition of bacterial growth as a function of treatment time.

## 4. Conclusions

*E. coli* biofilms grown on different supports were active in the biodegradation of nitrates from water resources. Nitrites were observed as intermediate degradation products, but they were also progressively evolved to nitrogen gas, being totally removed from solutions. The denitrification reaction was faster for bacteria biofilms supported on carbon phases than on metal oxide supports, as follows: M500 > SiO_2_ > TiO_2_ > Al_2_O_3_. This trend suggested the best performance for biofilms was on highly porous and non-acidic supports. Carbon–metal oxide composites (C/SiO_2_, C/TiO_2_, and C/Al_2_O_3_) were synthesized to determine the influence of the inorganic phase’s nature on the performance of supports. Composites typically show an intermediate performance between pure phases following the same tendency. The formation of large mesopores and macropores favored well-adhered colonies. SEM analysis showed a larger EPS segregation when biofilms were supported on materials containing TiO_2_ and mainly Al_2_O_3_, denoting a less favorable environment. The worst performance of alumina was associated with the Brönsted character of their acid sites, because *E. Coli* are Gram-negative bacteria. With the formation of these composites, the thermoreduction of these stronger acidic sites by the organic phase during carbonization favors the performance of composites regarding pure metal oxides. Toxicity analysis with *Vibrio fischeri* also indicated a larger reduction in luminescence using acidic supports. Total nitrate and nitrite reduction were observed in the batch reactors; NO_3_^−^ reduction is faster and preferential considering the intermediate NO_2_^−^ being progressively delayed when the acidic character of the support is increased.

## Figures and Tables

**Table 1:** Toxicity determined according to ISO 11348−2:2007 for all materials used as bacteria supports.

Toxicity	M500	SiO_2_	TiO_2_	Al_2_O_3_	SiO_2_/C	TiO_2_/C	Al_2_O_3_/C
I_15_ (%)	7.1	7.1	25.4	46.0	8.4	19.7	39.5
I_30_ (%)	10.4	11.8	26.6	46.6	9.4	26.5	38.7

I_x_ (%) = Intensity after a 15- or 30-min exposure period.

**Table 2:** Textural characteristics and point of zero charge (pH_PZC_) of pure materials used as *E. coli* supports.

Support	pH_PZC_	S_BET_ (m^2^ g^−1^)	V_micro_ (cm^3^ g^−1^)	V_meso_ (cm^3^ g^−1^)	V_total_ (cm^3^ g^−1^)	L_0_ N_2_ (nm)
M500	6.3	611	0.285	1.035	1.320	1.63
SiO_2_	7.1	241	0.094	0.889	0.983	1.78
TiO_2_	4.7	116	0.047	0.446	0.493	1.89
Al_2_O_3_	4.3	121	0.047	0.197	0.244	1.61

Results obtained from N_2_ adsorption isotherms: S_BET_ = apparent surface area; V_micro_ = micropore volume; V_meso_ = mesopore volume; V_total_ = total pore volume; L_0_ N_2_ = mean micropore width accessible to N_2_ at −196 °C.

**Table 3:** Textural characteristics and pH_PZC_ of M500 and the carbon–metal oxide composites used as *E. Coli* supports.

Sample	Metal (%)	pH_PZC_	V_CO2_ (cm^3^ g^−1^)	V_meso_ (cm^3^ g^−1^)	V_macro_ (cm^3^ g^−1^)	*ρ*_p_ (g cm^−3^)	S_ext_ (m^2^ g^−1^)	S_CO2_ (m^2^ g^−1^)
M500	−−	6.3	0.171	0.613	0.000	0.76	185	513
C/SiO_2_	42.0	6.0	0.154	0.033	0.070	1.24	15	407
C/TiO_2_	49.0	6.7	0.145	0.000	0.249	1.02	12	382
C/Al_2_O_3_	47.0	7.3	0.160	0.265	0.799	0.58	60	421

Metal = metal oxide content (wt%); V_CO2_ = micropore volume determined from CO_2_ isotherms; V_meso_ = mesopore volume; V_macro_ = macropore volume; *ρ*_p_ = bulk density; S_ext_ = external surface area; SCO_2_ = micropores surface area.

## Data Availability

Not applicable.

## Sample Availability

Samples of the composite materials and carbon gels are available from the authors, the rest of inorganic supports are available commercially.
